# Randomized clinical trials of COVID-19 vaccines: Do adenovirus-vector vaccines have beneficial non-specific effects?

**DOI:** 10.1016/j.isci.2023.106733

**Published:** 2023-04-25

**Authors:** Christine S. Benn, Frederik Schaltz-Buchholzer, Sebastian Nielsen, Mihai G. Netea, Peter Aaby

**Affiliations:** 1 Bandim Health Project, Indepth Network, Apartado 861, Bissau, Guinea-Bissau; 2 OPEN, Odense Patient Data Explorative Network, Institute of Clinical Research Odense University Hospital/ University of Southern Denmark, Odense, Denmark; 3 Danish Institute of Advanced Science, University of Southern Denmark, Odense, Denmark; 4 Department of Internal Medicine and Radboud Center for Infectious Diseases, Radboud University Medical Center, Nijmegen, the Netherlands; 5 Department of Immunology and Metabolism, Life and Medical Science Institute, University of Bonn, Bonn, Germany

**Keywords:** Microbiology, Virology

## Abstract

We examined the possible non-specific effects of novel mRNA- and adenovirus-vector COVID-19 vaccines by reviewing the randomized control trials (RCTs) of mRNA and adenovirus-vector COVID-19 vaccines. We calculated mortality risk ratios (RRs) for mRNA COVID-19 vaccines vs. placebo recipients and compared them with the RR for adenovirus-vector COVID-19 vaccine recipients vs. controls. The RR for overall mortality of mRNA vaccines vs. placebo was 1.03 (95% confidence interval [CI]: 0.63–1.71). In the adenovirus-vector vaccine RCTs, the RR for overall mortality was 0.37 (0.19–0.70). The two vaccine types differed significantly with respect to impact on overall mortality (p = 0.015). The RCTs of COVID-19 vaccines were unblinded rapidly, and controls were vaccinated. The results may therefore not be representative of the long-term effects. However, the data argue for performing RCTs of mRNA and adenovirus-vector vaccines head-to-head comparing long-term effects on overall mortality.

## Highlights

• It is unknown if COVID-19 vaccines have non-specific effects on overall survival
• The randomized trials of mRNA and adenovirus vaccines had information on deaths
• The relative mortality risk for mRNA vaccine vs. placebo was 1.03 (0.63–1.71)
• For adenovirus vaccine vs. placebo, it was significantly different: 0.37 (0.19–0.70)

## Graphical abstract

**Figure:**
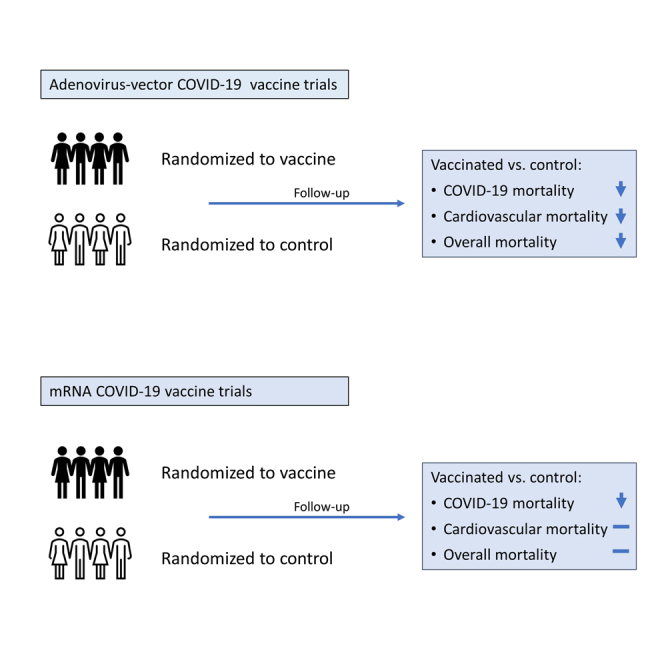

## Introduction

Within the current understanding of vaccines, it is logical to assume that COVID-19 vaccines reduce overall mortality corresponding to the number of COVID-19 deaths prevented. However, there is now ample evidence that vaccines can have broad non-specific effects on the immune system.^1^^,^^2^ These effects can lead to additional protection or increased susceptibility to unrelated infections and non-infectious immune-mediated diseases.^3^ Therefore, as it has now been established in numerous studies, vaccines may have effects on overall mortality that are different from what could be anticipated based on the protection against the vaccine-targeted microorganism.

The current system for testing vaccines does not incorporate this possibility: when the new COVID-19 vaccines were tested in randomized clinical trials (RCTs) against placebo/control vaccine, the trials were not designed to assess the effect on overall mortality. The possibility for observing such effects was further hampered by the short follow-up in these trials as the individuals from the control groups were offered vaccination after 2–6 months, following the emergency use authorization. Hence, though it was anticipated that the new COVID-19 vaccines would reduce overall mortality, especially in the context of a pandemic, this has not been formally studied.

Given the public health importance, we used the final study reports currently available from the clinical testing to examine the impact of mRNA and adenovirus-vector COVID-19 vaccines on overall mortality, including COVID-19-related mortality, accidents, cardiovascular deaths, and the category of deaths that would represent the deaths that would likely be affected by non-specific effects of vaccines, namely “non-accident, non-COVID-19” mortality.

## Results

We identified three RCTs of mRNA vaccine^4^^,^^5^^,^^6^ and six RCTs of adenovirus-vector COVID-19 vaccines^7^^,^^8^^,^^9^^,^^10^^,^^11^^,^^12^^,^^13^ with mortality data available (Table S1).

### mRNA vaccines

The two major RCTs of mRNA vaccines, produced by Pfizer and Moderna, included 74,193 adults (>16 or >18 years of age) (37,110 vaccinated; 37,083 placebo), among whom there were 61 deaths (31 vaccine recipients, 30 placebo recipients).^4^^,^^5^ These vaccines were not associated with lower overall mortality, the overall RR being 1.03 (0.63–1.71) (Table 1). The third RCT of mRNA vaccine, from CureVac,^6^ reported 8 deaths in the vaccine group and 6 in the placebo group, suggesting a similar trend as in the other two mRNA trials. However, the deaths in the CureVac RCT were not reported by cause of death, and since the vaccine has now been withdrawn due to low vaccine efficacy, it has not been included in Table 1.

**Table 1:** Overall and non-COVID-19 mortality in the RCTs of mRNA vaccines

	Vaccine group (deaths/N)	Placebo group (deaths/N)	Relative risk (95% CI)
**Pfizer vs. placebo**^4^			

Overall mortality	15/21926	14/21921	1.07 (0.52–2.22)
COVID-19 mortality	1/21926	2/21921	0.50 (0.05–5.51)
Cardiovascular mortality^a^	9/21926	6/21921	1.50 (0.53–4.21)
Other non-COVID-19 mortality	5/21926	5/21921	1.00 (0.29–3.45)
Accident mortality^b^	0/21926	1/21921	0.0
Non-accident, non-COVID-19 mortality	14/21926	11/21921	1.27 (0.58–2.80)

**Moderna vs. placebo**^5^			

Overall mortality	16/15184	16/15162	1.00 (0.50–2.00)
COVID-19 mortality	1/15184	3/15162	0.33 (0.03–3.20)
Cardiovascular mortality^a^	7/15184	5/15162	1.40 (0.44–4.40)
Other non-COVID-19 mortality	6/15184	7/15162	0.86 (0.29–2.55)
Accident mortality^c^	2/15184	1/15162	2.00 (0.18–22.02)
Non-accident, non-COVID-19 mortality	13/15184	12/15162	1.08 (0.49–2.37)

**Combined for Pfizer and Moderna vs. placebo**^d^			

Overall mortality	31/37110	30/37083	1.03 (0.63–1.71)
COVID-19 mortality	2/37110	5/37083	0.40 (0.08–2.06)
Cardiovascular mortality	16/37110	11/37083	1.45 (0.67–3.13)
Other non-COVID-19 mortality	11/37110	12/37083	0.92 (0.40–2.08)
Accidents	2/37110	2/37083	1.00 (0.14–7.09)
Non-accident, non-COVID-19 mortality	27/37110	23/37083	1.17 (0.67–2.05)

Notes: In the Pfizer and Moderna trials there were discrepancies between the number of deaths presented in the flowchart and in the text/tables; we have used the numbers in the text/tables. The Pfizer study could have more than one cause ascribed to a death; there were a total of 17 diagnoses among the 15 deaths in the vaccinated group and 17 diagnoses among 14 deaths in the placebo group; thus, some overlap cannot be excluded.

a Judged as cardiovascular deaths: from the Pfizer trial: “Myocardial infarction” (N = 2); “Hypertensive heart disease” (N = 1); “Hemorrhage stroke” (N = 1); “Cardiorespiratory arrest” (N = 2); “Cardiac failure congestive” (N = 1); “Cardiac arrest” (N = 5); “Arteriosclerosis” (N = 2); “Aortic rupture” (N = 1). From the Moderna trial: “Myocardial infarction” (N = 5) “Cardiopulmonary arrest” (N = 3); “End-stage congestive heart failure” (N = 1); “Cardiac arrest” (N = 1); “Provisional diagnosis, sudden fatal event, likely myocardial infarction” (N = 1); “Death suspected due to coronary heart disease, probably due to complications of diabetes mellitus” (N = 1).

b There was one death due to an overdose.

c There were three deaths due to suicide (2) and head trauma (1).

d Mantel-Haenszel estimate.

Though not statistically significant, the Pfizer^4^ and Moderna^5^ vaccines were associated with a lower risk of COVID-19 death (RR = 0.40 (0.08–2.06)) (Table 1). As expected, they were not associated with the risk of accidental deaths. Fifty percent of the non-COVID-19 deaths (27/54) were cardiovascular; there was no beneficial effect of the vaccines on such deaths (RR = 1.45 (0.67–3.13)). The RR for non-accident, non-COVID-19 death was 1.17 (0.67–2.05) (Table 1).

### Adenovirus-vector vaccines

The five RCTs of adenovirus-vector COVID-19 vaccines presented in Table 2 included 122,164 adults (72,138 vaccinated; 50,026 controls) among whom there were 46 deaths (16 vaccine recipients, 30 controls). A subsequent paper^14^ for the Johnson&Johnson vaccine provided data on longer follow-up (121 days), but information on non-COVID-19 causes of death was not provided for the present analysis; hence, we used the data from the previous report (see sensitivity analysis). The interim report of the Ad5-nCoV RCT^13^ did not report the deaths and causes of death by randomization group and was not included in the analysis.

**Table 2:** Overall and non-COVID-19 mortality in the RCTs of adenovirus-vector vaccines

	Vaccine group (deaths/N)	Placebo group (deaths/N)	Relative risk (95% CI)
**AstraZeneca vs. placebo US/Chile/Peru**^7^^a^			

Overall mortality	7/21587	7/10792	0.50 (0.18–1.42)
COVID-19 mortality	0/21587	2/10792	0.0
Cardiovascular mortality^b^	0/21587	2/10792	0.0
Other non-COVID-19 mortality	3/21587	3/10792	0.50 (0.10–2.48)
Accidents^c^	4/21587	0/10792	N/A
Non-accident, non-COVID-19 mortality	3/21587	5/10792	0.30 (0.07–1.25)

**AstraZeneca vs. placebo South-Africa**^8^^,^^9^			

Overall mortality	1/1011	3/1010	0.33 (0.03–3.20)
COVID-19 mortality	0/1011	0/1010	N/A
Cardiovascular mortality	0/1011	0/1010	N/A
Other non-COVID-19 mortality	0/1011	0/1010	N/A
Accidents^d^	1/1011	3/1010	0.33 (0.03–3.20)
Non-accident, non-COVID-19 mortality	0/1011	0/1010	N/A

**AstraZeneca vs. control vaccine UK, Brazil**^10^			

Overall mortality	2/11218	3/10901	0.65 (0.11–3.88)
COVID-19 mortality	0/11218	1/10901	0.0
Cardiovascular mortality	0/11218	0/10901	N/A
Other non-COVID-19 mortality	2/11218	1/10901	1.94 (0.18–21.43)
Accidents^e^	0/11218	1/10901	0.0
Non-accident, non-COVID-19 mortality	2/11218	1/10901	1.94 (0.18–21.43)

**Johnson&Johnson vs. placebo**^11^			

Overall mortality	3/21895	16/21888	0.19 (0.05–0.64)
COVID-19 mortality	0/21895	5/21888	0.0
Cardiovascular mortality^b^	0/21895	2/21888	0.0
Other non-COVID-19 mortality	3/21895	7/21888	0.43 (0.11–1.66)
Accidents^f^	0/21895	2/21888	0.0
Non-accident, non-COVID-19 mortality	3/21895	9/21888	0.33 (0.09–1.23)

**Gam-COVID-Vac vs. placebo**^12^			

Overall mortality	3/16427	1/5435	0.99 (0.10–9.54)
COVID-19 mortality	2/16427	0/5435	N/A
Cardiovascular mortality^b^	0/16427	1/5435	0.0
Other non-COVID-19 mortality	0/16427	0/5435	N/A
Accidents^g^	1/16427	0/5435	N/A
Non-accident, non-COVID-19 mortality	0/16427	1/5435	0.0

**Combined**^h^			

Overall mortality	16/72138	30/50026	0.37 (0.19–0.70)
COVID-19 mortality	2/72138	8/50026	0.11 (0.02–0.87)
Cardiovascular mortality	0/72138	5/50026	0.065 (0.01–0.41)^i^
Other non-COVID-19 mortality	8/72138	11/50026	0.58 (0.23–1.45)
Accidents	6/72138	6/50026	0.69 (0.19–2.58)
Non-accident, non-COVID-19 mortality	8/72138	16/50026	0.38 (0.17–0.88)

Notes: In the AstraZeneca paper, data from UK and Brazil trials, using meningitis vaccine in the control group, were published in a meta-analysis with data from a South Africa trial,^8^^,^^9^ which used placebo. The South African trial has also been reported separately. We maintained the division in placebo and control vaccine. The RCT of Ad5-nCoV^13^ vaccine did not present causes of death by randomization group.

a A publication from this AstraZeneca RCT reports data until July 30, 2021.^38^ While this provided longer follow-up and more deaths, the unbiased comparison of vaccinated and controls was broken by unblinding and provision of non-study COVID-19 vaccines, resulting in severe skewing of the follow-up time in the safety population, with a median follow-up time to receipt of non-study COVID-19 vaccine of 228 days in the 21,587 participants in vaccinated group vs. 103 days in the 10,793 participants in the placebo group. Interestingly, during this period, there were 14 vs. 8 deaths. This corresponds to death rates of 0.28/100,000 person days at risk vs. 0.72/100,000 person days at risk giving a relative risk of 0.40 (0.17–0.94), supporting a strong beneficial effect of the AstraZeneca vaccine. However, due to the unblinding, this result has not been included in the present analysis.

b Judged as cardiovascular deaths: from AstraZeneca: “cardiac arrest”; “hemorrhagic transformation stroke” + “ischemic stroke”. From Johnson&Johnson trial: “acute myocardial infarction”; “cardiac failure”. From Gam-COVID-Vac trial: “hemorrhage stroke”.

c There were four deaths: overdose (2), accident, road traffic accident.

d There were four deaths: gunshot, blunt force trauma to head, homicide, suicide.

e There was one death: traumatic brain injury.

f There were two deaths: overdose, suicide.

g There was one death: fracture of thoracic vertebra.

h Mantel-Haenszel estimate.

i Due to 0 events among the vaccinated we used the Peto OR method for pooling trial results.

The adenovirus-vector vaccines were associated with a reduction in overall mortality, the RR being 0.37 (0.19–0.70) (Table 2). This was due to lower COVID-19 mortality (RR = 0.11 (0.02–0.87)) and lower cardiovascular mortality (0 vs. 5 deaths, Table 2).

The trend for lower overall mortality was consistent in the larger trials of AstraZeneca^7^^,^^8^^,^^9^^,^^10^ (combined RR for the three AstraZeneca trials = 0.50 (0.22–1.15)) and Johnson&Johnson^11^ (RR = 0.19 (0.05–0.64)), but not in the Gam-COVID-Vac RCT,^12^ which had only four deaths. The AstraZeneca RCTs in UK and Brazil^10^ used a control vaccine rather than a placebo, but there were too few events to examine whether that had an impact.

Twenty-six percent (12/46) of deaths in the adenovirus-vector vaccines RCTs were due to accidents. The non-accident, non-COVID-19 RR was 0.38 (0.17–0.88) in the adenovirus-vector RCTs (0.33 (0.09–1.23) for Johnson&Johnson, 0.52 (0.17–1.60) for AstraZeneca) (Table 2).

### Comparison of mRNA and adenovirus-vector vaccines

**Figure 1:**
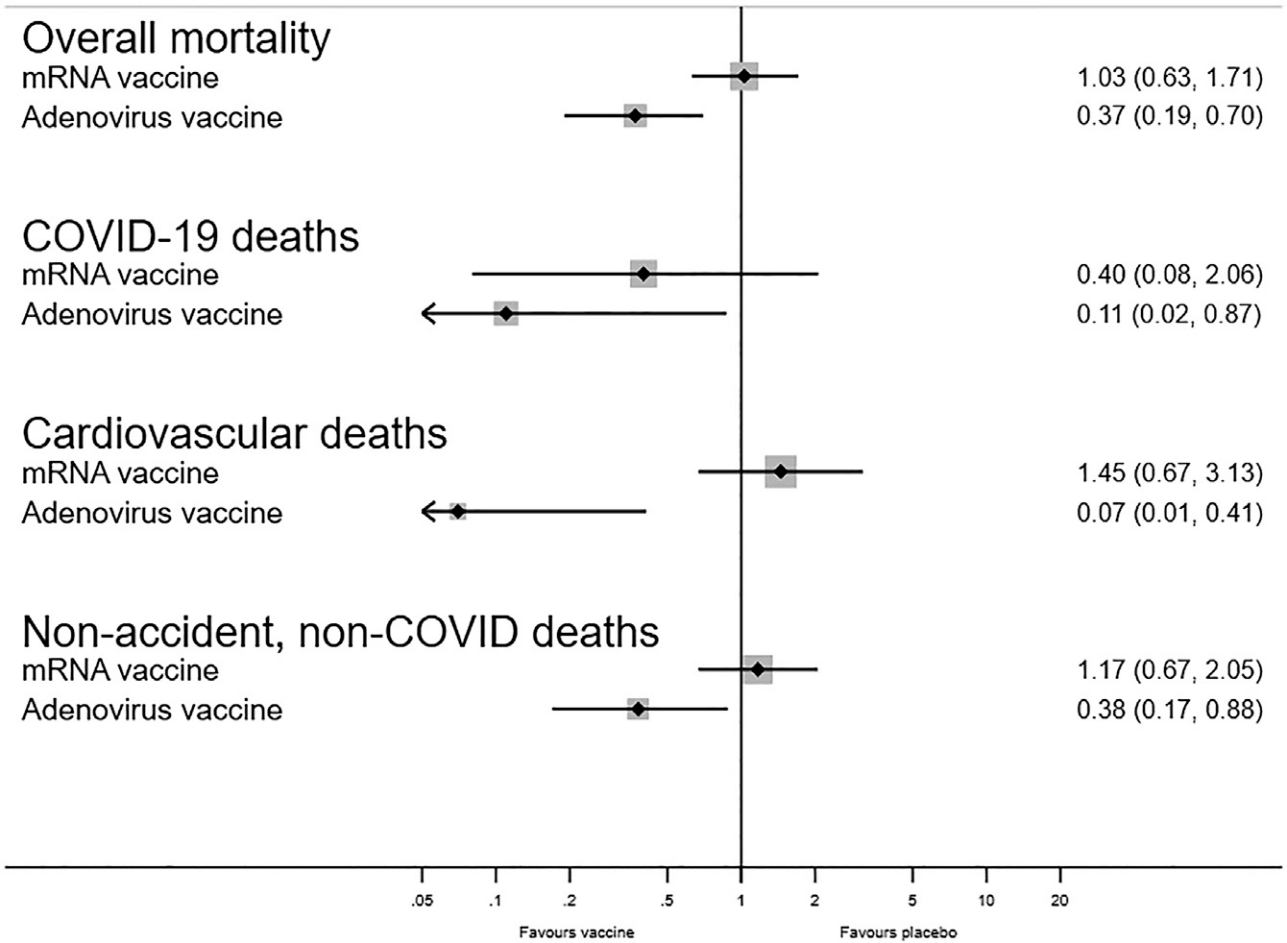
Forest plot comparing estimated effects of mRNA COVID-19 vaccines versus placebo and of adenovirus-vector COVID-19 vaccines versus placebo/control vaccine with respect to impact on overall mortality, COVID-19 mortality, cardiovascular death, and non-accident, non-COVID-19 mortality

Both types of vaccines protected against COVID-19 death but had differential effects on overall mortality (p = 0.015) (Figure 1). Compared with the mRNA RCTs, the RR for overall mortality was lower in the adenovirus RCTs, with a ratio-ratio of 0.36 (0.16–0.82) ((0.37 (0.19–0.70)/1.03 (0.63–1.71)) (Tables 1 and 2). The two groups of vaccines also differed with respect to “non-accident, non-COVID-19 mortality” (test of homogeneity, p = 0.027). The impact differed most strongly for cardiovascular deaths (p = 0.002) (Tables 1 and 2). Compared with the mRNA vaccines (Table 1), both Johnson&Johnson (p = 0.016) and AstraZeneca (p = 0.14) tended to have lower overall mortality.

### Sensitivity analyses

If the underlying mortality rates for different causes of death differed in the RCTs, this could affect the comparison between mRNA and adenovirus-vector vaccines. In the unvaccinated control groups, accidents and COVID-19 deaths tended to be more common in the adenovirus-vector trials than in the mRNA trials; but these deaths were few, and there was no difference in the rates of other deaths and overall mortality in the mRNA and adenovirus-vector vaccine trials (Table S2).

Using the overall mortality data from the longer follow-up (121 days) for the Johnson&Johnson vaccine,^14^ with 28 deaths in the vaccine group and 55 in the control group (5 and 22, respectively, due to COVID-19), the combined RR for overall mortality in the adenovirus-vector RCTs became 0.52 (0.35–0.77), a significant difference from the effect in the mRNA RCTs (p = 0.035).

## Discussion

### Principal findings

In the RCTs with the longest possible blinded follow-up, mRNA vaccines had no effect on overall mortality despite protecting against some COVID-19 deaths. On the other hand, the adenovirus-vector vaccines were associated with lower overall mortality. The adenovirus-vector vaccines were further associated with a lower risk of dying from the causes of death that would most likely represent non-specific effects of vaccines, the “non-accident, non-COVID-19” deaths. The pattern of effects was internally consistent within the RCTs of mRNA and adenovirus-vector vaccines.

An intrinsic limitation for the estimation of overall mortality during the COVID-19 pandemic is the nature of the cohorts studied. Most of the volunteers participating in the trials were adult individuals in general good health, resulting in low COVID-19 and overall mortality. In a real-life situation in which the COVID-19 vaccines are administered to highly vulnerable populations with high COVID-19-related mortality, significant reductions in overall mortality are expected, also for mRNA vaccines. However, the intriguing differences in the effects on non-accident, non-COVID-19 mortality are likely to persist and should be investigated in future studies.

The results suggest that adenovirus-vector vaccines compared with placebo have beneficial non-specific effects, reducing the risk of non-COVID-19 diseases. The most important cause of non-COVID-19 death was cardiovascular disease, against which the data for the current RCTs suggest that the adenovirus-vector vaccines provide at least some protection.

### Potential sources of bias in the comparison of the two vaccine types

Differences between the study populations in the RCTs of the two vaccine types could have biased the comparison as different disease patterns and level of care could have influenced the measured effect of the vaccines on overall mortality. A slightly larger proportion of the participants from the adenovirus RCTs may have been from middle- and low-income countries (Table S1). More individuals were infected with COVID-19 in the mRNA RCTs than in the adenovirus-vector vaccine RCTs (Table S1), but there were more COVID-19 deaths in the adenovirus-vector RCTs (Table S2). This suggests that participants in the mRNA RCTs may have had access to better health care during COVID-19 infection, and this may have reduced the impact of mRNA vaccination on overall mortality. There was a higher proportion of accidents in the adenovirus-vector vaccine RCTs, and when accidents were excluded, the contrast between mRNA and adenovirus-vector vaccines became more pronounced. Cardiovascular deaths were more common in the mRNA RCTs; participants in these trials may have had more co-morbidities or more events because they had longer follow-up (Table S1). However, the effect of COVID-19 vaccines on cardiovascular events differed, being possibly beneficial for the adenovirus-vector vaccines but not for the mRNA vaccines. The lack of impact of mRNA vaccines on cardiovascular morbidity is supported by a recent epidemiological survey in France.^15^ Altogether, it is unlikely that differences in mortality patterns in the RCTs of the mRNA and the adenovirus-vector vaccines can fully explain the apparent contrast in overall mortality effects between the two types of vaccines.

### Subtypes of mRNA vaccines and adenovirus-vector vaccines

The Pfizer and Moderna mRNA vaccines are technologically very similar.^16^ However, there are subtle differences between the two vaccines, both with respect to the RNA and the carriers and the amount of RNA per dose. The present analysis had low power to detect differences in effect between the two mRNA vaccines.

The different adenovirus-vector vaccines are based on different adenovirus vectors.^16^ Pre-existing immunity against the vector may thus differ between the various vaccines. Furthermore, different vaccine technologies were applied.^16^ Only the Johnson&Johnson and the AstraZeneca vaccines were examined in studies large enough to assess differences in mortality; these two vaccine types appeared to have similar beneficial non-specific effects on overall mortality.

### Comparison with other studies

Apart from the RCTs presented here, the overall mortality effects of the two vaccine types have only been evaluated in observational studies. Particular beneficial effects on overall mortality of AstraZeneca and Gam-COVID-Vac, albeit not Johnson&Johnson adenovirus-vector vaccines, in comparison with Pfizer and Moderna mRNA vaccines were seen in a Hungarian observational study.^17^ Beneficial effects on overall mortality were reported in an observational study from Buenos Aires in Argentina, which mainly used non-mRNA vaccines.^18^ In contrast to the RCTs of mRNA vaccines, an observational study from Center for Disease Control (CDC)^19^ reported lower rates of non-COVID-19 mortality among mRNA-vaccinated individuals. However, these observational studies have numerous sources of bias, including healthy vaccinee bias, and merely underscore that RCTs are needed to assess the association between vaccination and overall health.

### Interpretation and immunological mechanisms

The substantial mortality reduction associated with adenovirus-vector vaccines appears difficult to understand if the expectation is that vaccines only protect against death from the target disease. The result may be interpreted as implausible and dismissed.^20^ It is therefore important to take into consideration that such non-specific effects, and their immunological basis, have been established for several other vaccines.^2^^,^^21^ Non-specific effects of vaccines have been observed to differ between live-attenuated vaccines and non-live vaccines; live vaccines have been associated with reduced all-cause mortality while this has not been seen for non-live vaccines.^2^ For example, RCTs have shown that Bacille Calmette Guérin (BCG) vaccine against tuberculosis reduces neonatal mortality by more than a third,^22^^,^^23^ due to protection against deaths from sepsis and respiratory infections. In parallel, immunological studies have provided a mechanism by showing that BCG induces “trained immunity” leading to enhanced resistance toward a broad range of pathogens.^21^^,^^24^ Interestingly, BCG vaccine has been associated with decreased systemic inflammation,^25^^,^^26^ which could in turn impact positively the outcome of diseases mediated by inflammatory processes (including cardiovascular diseases).

For the AstraZeneca vaccine it was recently shown that monocyte frequency and count were increased up to 3 months after vaccination compared with their pre-vaccine levels.^27^ Monocytes exhibited enhanced antigen presentation functions and had increased capacity to produce key cytokines and chemokines in response to unrelated stimuli. Thus, the vaccine seems to induce trained immunity.^27^ We speculate that, even though the adenovirus-vector is replication deficient, it may prime the immune system in a way similar to a “live” vaccine. A reduced risk of infections would be anticipated to lead to lower risk of overall mortality, including a lower risk of cardiovascular deaths,^28^ just as seen for influenza vaccination.^29^

A recent study has reported that the Pfizer mRNA vaccines modulate transcriptional profiles in innate immune cells.^30^ Another study showed that the vaccine modulated the production of inflammatory cytokines by innate immune cells upon stimulation with non-specific stimuli.^31^ However, the impact on the antimicrobial functions of these cells is not yet known. In an animal study, the lipid nanoparticles carrying the vaccine were associated with enhanced inflammation.^32^

After their introduction, the adenovirus-vector vaccines were linked to an increased risk of cardiovascular events.^33^ However, more recently, there have also been reports of such increased risk after mRNA vaccination,^34^ and both vaccine types have been associated with enhanced platelet activation, albeit more so for the adenovirus-vector vaccines than the mRNA vaccines.^35^

Taken together, the “trained immunity” induced by adenovector-vaccines and the enhanced inflammation after mRNA vaccines could help explain their contrasting effects on overall mortality, including cardiovascular deaths. It should be noted, though, that the trained immunity phenotype induced by endogenous mediators (such as oxLDL or Lp(a)) might be linked to the development of atherosclerosis;^36^ so studies with longer follow-up than just a few months would be important to determine the net effect.

### Implications

Marked differences in the overall mortality impact between two of the major types of COVID-19 vaccines used in the world are of obvious public health importance.

While mass-vaccination programs with COVID-19 vaccines are rolled out, data on their effects on non-COVID-19 mortality should be collected. As COVID-19 mortality comes under better control due to herd immunity and increasing vaccination coverage, the impact on non-COVID-19 mortality becomes particularly important from a public health perspective.

Unfortunately, the opportunity for conducting large-scale RCTs vaccine-vs.-placebo trials passed once the vaccines were introduced generally in the population. To throw light on the potential differences in non-specific effects between vaccine types, an obvious way forward would be to conduct RCTs comparing the mRNA vaccines and adenovirus-vector vaccines for their effect on COVID-19 mortality, as well as non-COVID-19 mortality. Even if effects within the group of adenovirus-vector vaccines would turn out to be more heterogeneous with longer follow-up and when more studies have been conducted, it seems clear that the overall health effects of the Johnson&Johnson and AstraZeneca vaccines should be tested against the leading mRNA vaccines. In addition, future trials of new COVID-19 vaccines should be compelled to report overall mortality data by cause, sex, and age. Post-licensure monitoring and evaluation should also focus on overall, non-accidental mortality.

### Conclusions

The differences in the effects of adenovirus-vector and mRNA vaccines on overall mortality, if true, would have a major impact on global health. If validated in additional studies, the protective non-specific effects of adenovirus-based vaccines on non-COVID-19 mortality, in addition to their effectiveness against severe acute respiratory syndrome coronavirus 2 (SARS-CoV-2) infection, may represent an important advantage in vulnerable populations in which cardiovascular mortality is high.

Ironically, the rich countries in Europe and the USA have emphasized the more expensive mRNA vaccines because of slightly better short-term vaccine efficacy against COVID-19 compared to the relatively inexpensive adenovirus-vector vaccines and the detection of a rare blood clotting disorder associated with the adenovirus-vector vaccines, mainly AstraZeneca. While this decision is understandable in the short term during a situation with high COVID-19-related mortality, in the endemic situation in which COVID-19-related deaths have decreased, this decision may need to be reassessed. Otherwise, if the protective effects of adenovirus-vector vaccines on overall mortality in the RCTs reflect the reality, this could turn out to be a very costly decision, both economically and health wise.

### Limitations of the study

The number of deaths in these RCTs was limited, and chance could therefore have played a role in these findings. However, the internally consistent effects and the large difference in effect sizes between the two vaccine types speak against “chance” as the main explanation.

Since there are well-established sex and age differences in the immune system, it is important to report and analyze data by sex and age group.^37^ This was unfortunately not possible for the present study since the RCTs only reported deaths by randomization allocation, not by sex and age.

## STAR★Methods

### Key resources table

**Table:** 

REAGENT or RESOURCE	SOURCE	IDENTIFIER
**Deposited data**		

Data from already published trials	We searched PubMed for RCTs with >1,000 participants of mRNA and adenovirus-vector COVID-19 vaccines and the web-site: https://covid19.trackvaccines.org/vaccines/approved/ for approved COVID-19 vaccines. We used the ClinicalTrials registry (https://clinicaltrials.gov/) to assess the status of each trial identified.	https://pubmed.ncbi.nlm.nih.gov/?term=randomised+controlled+trial+covid-19+vaccine+&sort=date https://covid19.trackvaccines.org/vaccines/approved/ Specific references are provided in the reference list
Data provided directly from authors of published trials	Authors	N/A

### Resource availability

#### Lead contact

Further information about resources should be directed to and will be fulfilled by the lead contact, Christine Stabell Benn (cbenn@health.sdu.dk).

#### Materials availability

All the material used for this paper were available in the published literature, except from where it is indicated that information was acquired directly from authors.

In January 2023, we searched PubMed for RCTs of mRNA and adenovirus-vector COVID-19 vaccines and the web-site: https://covid19.trackvaccines.org/vaccines/approved/ for approved COVID-19 vaccines.

### Method details

For all registered RCTs for mRNA and adenovirus-vector vaccines with more than 1,000 participants, we used the registration number to search for related publications. For each trial we also used the trial registration number to identify the most recent report. Most RCTs used a placebo, but as indicated in Table S1, a few RCTs used a control vaccine. RCTs of adolescents and children were not included.

The RCTs reported deaths as part of the safety assessment. We grouped non-health-related deaths due to suicide, homicide, overdose, trauma, and traffic accidents as “accidents”. Apart from COVID-19 deaths and accidents, a large part of non-COVID-19 deaths were due to cardiovascular diseases. We therefore analysed overall mortality in the following categories: COVID-19 deaths, accidents, cardiovascular deaths, and other non-COVID-19 deaths. The two last groups were also combined in the category “non-accident, non-COVID-19 deaths”, which covered deaths of natural causes other than COVID-19.

Several RCTs did not report the deaths by randomization group or in sufficient detail to distinguish between these categories. In these cases, we wrote to the main authors to ask for the missing information. The additional information obtained in this way is described in Acknowledgements.

### Quantification and statistical analysis

In the main analyses, we examined the mortality risk rather than the mortality rate, as follow-up time was not reported in a consistent manner. Follow-up was reported as median or mean days of follow-up from 1^st^ vaccination, from 2^nd^ vaccination, or from 7 to 14 days after 2^nd^ vaccination (Table S1). Sometimes the main trial focus had been on the individuals who were seronegative at enrolment, while sometimes the safety data-set covered everybody who had been enrolled in the trial. To assess the approximate follow-up time in the different trials and the crude mortality rates by 10,000 person-years (pyrs), we estimated the days of follow-up in the control groups from the 1^st^ vaccination in the safety data-set; in trials which did not report time from first vaccination but from the 2^nd^ vaccination we assumed that participants followed the prescribed number of days between the 1^st^ and 2^nd^ dose of vaccine (Table S2).

The mortality data from several studies were combined using the Mantel-Haenszel estimator providing risk ratios (RRs).

## Data Availability

This paper does not use original data or original code. Any additional information required to reanalyze the data reported in this paper is available from the lead contact upon request.
