# Supplementary material for: Randomized clinical trials of COVID-19 vaccines: Do adenovirus-vector vaccines have beneficial non-specific effects?
Source: iScience. 2023 Apr 25;26(5):106733. doi: 10.1016/j.isci.2023.106733 (PMC10125209; doi:10.1016/j.isci.2023.106733)
Supplement: Document S1. Tables S1 and S2 [file mmc1.pdf]

iScience, Volume *26*

Supplemental information

Randomized clinical trials of COVID-19 vaccines: Do adenovirus-vector vaccines have beneficial non-specific effects?

Christine S. Benn, Frederik Schaltz-Buchholzer, Sebastian Nielsen, Mihai G. Netea, and Peter Aaby

Randomized clinical trials of COVID-19 vaccines:

do adenovirus-vector vaccines have beneficial non-specific effects?

Christine S. Benn, DMSc[1,2,3], Frederik Schaltz-Buchholzer, MD, PhD[1,2], Sebastian Nielsen, MSc, PhD[1,2], Mihai G. Netea, MD, PhD[4,5], Peter Aaby, DMSc[1,2]

[1]Bandim Health Project, Indepth Network, Apartado 861, Bissau, Guinea-Bissau

[2]OPEN, Odense Patient data Explorative Network, Institute of Clinical Research; Odense University Hospital/ University of Southern Denmark, Denmark

[3]Danish Institute of Advanced Science, University of Southern Denmark, Denmark

[4]Department of Internal Medicine and Radboud Center for Infectious Diseases, Radboud University Medical Center, Nijmegen, Netherlands

[5]Department of Immunology and Metabolism, Life and Medical Science Institute, University of Bonn, Bonn, Germany

Lead contact: Christine S. Benn, cbenn@health.sdu.dk

Table S1. Background information about included RCTs, Related to tables 1 and 2.

	Pfizer [1]	Moderna [2]	CVnCoV [3]	AZ US/Chile/Peru [4]	AZ South Africa [5,6]	AZ UK/Brazil [7]	Johnson&Johnson [8]	Gam-COVID-Vac [9]	Ad5-nCoV [10]
**Trial registration number**	NCT04368728	NCT04470427	NCT04652102	NCT04516746	NCT04444674	ISRCTN89951424 NCT04324606 NCT04400838	NCT04505722	NCT04530396	NCT04526990
**N**	44,165	30,415	39,680	32,451	2021	22119	39,321	21,977	36,717
**Vaccine**	mRNA	mRNA	mRNA	Chimpanzee Adenovirus Y25	Chimpanzee Adenovirus Y25	Chimpanzee Adenovirus Y25	Human Adenovirus 26	Human Adenovirus 26 and 5	Human Adenovirus 5
**No of doses**	2	2	2	2	2	2	1	2	1
**Phase**	2/3	3	2b/3	3	1/2	1/2/3	3	3	3
**Randomization ratio**	1:1	1:1	1:1	2:1	1:1	1:1	1:1	3:1	1:1
**Intervention**	BNT162b2	mRNA-1273	CVnCoV	AZD1222	AZD1222	AZD1222	Ad26.COV2.S	Gam-COVID-Vac	Ad5-nCoV
**Control group**	Saline	Placebo[b]	Saline	Saline	Saline	Control vaccine (MenACWY)	Saline	Vaccine buffer	Vaccine buffer
**Vaccine efficacy[a]**	91% (89-93%)	93% (91-95%)	48% (31-61%)	74% (65-81%)	22% (-50-60%)	67% (57-74%)	67% (59-73%)	92% (86-95%)	58% (40-70%)
**Age enrolled (years)**	16+	18+	18+	18+	18-65	18+	18+	18+	18+
**Male %**	51	53	55	56	57	42	55	61	71
**Age (mean or median (range))**	51 (16-91)	51 (18-95)	43 (IQR 31-54)	50 (18-100)	30 (18-64)	83%<55years	52 (18-100)	45 (SD 12)	38 (18-94)
**Geographical location**	US, Argentina, Brazil, South Africa, Germany, Turkey	US	Belgium, Germany, Netherlands, Spain, Argentina, Colombia, Dominican	US, Chile, Peru	South Africa	UK, Brazil	US, Argentina, Brazil, Chile, Colombia, Mexico, Peru, South Africa	Russia	Argentina, Chile, Mexico, Pakistan, Russia

			Republic, Mexico, Panama, Peru						
**Proportion recruited in the US**	76%	100%	0%	89%	0%	0%	44%	0%	0%
**Enrolment period and data cut-off**	July 27, to Oct 29, 2020 Data cut-off March 13, 2021	July 27 to Oct 23, 2020 Data cut-off March 26, 2021	Dec 11, 2020 to April 12, 2021 Data cut-off June 18, 2021	Aug 28, 2020, to Jan 15, 2021 Data cut-off March 5,2021	June 24 and Nov 9, 2020	April 23 and Dec 6, 2020 Data cut-off Dec 6, 2020	Sept 21, 2020, to Jan 22, 2021 Data cut-off Jan 22, 2021	Sept 7 to Nov 24, 2020 Data cut-off Nov 24, 2020	Sept 22, 2020 to Jan 15, 2021 Data cut-off Jan 15, 2021
**Duration of follow-up**	Mean follow-up from 7 days after 2nd dose: 108 days	Median follow-up from 1st dose: 5.3 months	Mean follow-up from 15 days after 2nd dose: 48 days	Median follow-up from 2nd dose: 61 days	Median follow-up from 1st dose: 156 days	Mean follow-up from 1st dose: 4.2 months	Median follow-up: 58 days	Median follow-up from 1st dose: 48 days	Median follow-up from 28 days post-vaccination: 45 days
**Follow-up days in control group**	2,937,414	2,441,082	N/A	960,488	157,560	1,384,427	1,269,504	260,880	N/A
**COVID-19 infection in control group**	4.1% (850/20713)	5.3% (744/14164)	1.2% (145/12211)	1.5% (130/8550)	3.2% (23/717)	2.9% (248/8581)	1.8% (348/19544)	1.3% (62/4902)	1.5% (211/14586)
**Cardiovascular deaths: % of all deaths**	52% (15/29)	38% (12/32)	No information	14% (2/14)	0%	0%	11% (2/19)	25% (1/4)	No information
**COVID-19 death: % of all deaths**	10% (3/29)	13% (4/32)	No information	14% (2/14)	0%	20% (1/5)	26% (5/19)	50% (2/4)	No information

a.The calculations of vaccine efficacy differ with respect to the number of days after vaccination that observation is started

b.no information on preparation

Table S2. The rate of causes of death per 10,000 person-years (pyrs) in the control groups by type of vaccine, Related to Tables 1 and 2

Cause of death	Rate deaths per 10,000 pyrs (number of deaths/pyrs) in the control group		Relative risk (95% CI) mRNA vs Adenovirus-vector vaccine in the control group
	mRNA RCTs: 14,725.5 pyrs	Adenovirus-vector RCTs: 11,041 pyrs	
**COVID-19 deaths**	3.4 (5/14726)	7.2 (8/11041)	0.47 (0.15-1.43)
**Accidents**	1.4 (2/14726)	5.4 (6/11041)	0.25 (0.05-1.24)
**Non-accident, non-COVID-19 deaths**	15.6 (23/14726)	14.5 (16/11041)	1.08 (0.57-2.04)
**Overall mortality**	20.4 (30/14726)	27.2 (30/11041)	0.75 (0.45-1.24)

References

1. Thomas, S.J., Moreira, E.D., Jr., Kitchin, N., Absalon, J., Gurtman, A., Lockhart, S., Perez, J.L., Perez Marc, G., Polack, F.P., Zerbini, C., et al. (2021). Safety and Efficacy of the BNT162b2 mRNA Covid-19 Vaccine through 6 Months. N Engl J Med *385*, 1761-1773. 10.1056/NEJMoa2110345.
2. El Sahly, H.M., Baden, L.R., Essink, B., Doblecki-Lewis, S., Martin, J.M., Anderson, E.J., Campbell, T.B., Clark, J., Jackson, L.A., Fichtenbaum, C.J., et al. (2021). Efficacy of the mRNA-1273 SARS-CoV-2 Vaccine at Completion of Blinded Phase. N Engl J Med *385*, 1774-1785. 10.1056/NEJMoa2113017.
3. Kremsner, P.G., Ahuad Guerrero, R.A., Arana-Arri, E., Aroca Martinez, G.J., Bonten, M., Chandler, R., Corral, G., De Block, E.J.L., Ecker, L., Gabor, J.J., et al. (2022). Efficacy and safety of the CVnCoV SARS-CoV-2 mRNA vaccine candidate in ten countries in Europe and Latin America (HERALD): a randomised, observer-blinded, placebo-controlled, phase 2b/3 trial. Lancet Infect Dis *22*, 329-340. 10.1016/S1473-3099(21)00677-0.
4. Falsey, A.R., Sobieszczyk, M.E., Hirsch, I., Sproule, S., Robb, M.L., Corey, L., Neuzil, K.M., Hahn, W., Hunt, J., Mulligan, M.J., et al. (2021). Phase 3 Safety and Efficacy of AZD1222 (ChAdOx1 nCoV-19) Covid-19 Vaccine. N Engl J Med *385*, 2348-2360. 10.1056/NEJMoa2105290.
5. Madhi, S.A., Baillie, V., Cutland, C.L., Voysey, M., Koen, A.L., Fairlie, L., Padayachee, S.D., Dheda, K., Barnabas, S.L., Bhorat, Q.E., et al. (2021). Efficacy of the ChAdOx1 nCoV-19 Covid-19 Vaccine against the B.1.351 Variant. N Engl J Med *384*, 1885-1898. 10.1056/NEJMoa2102214.
6. Madhi, S.A., Koen, A.L., Izu, A., Fairlie, L., Cutland, C.L., Baillie, V., Padayachee, S.D., Dheda, K., Barnabas, S.L., Bhorat, Q.E., et al. (2021). Safety and immunogenicity of the ChAdOx1 nCoV-19 (AZD1222) vaccine against SARS-CoV-2 in people living with and without HIV in South Africa: an interim analysis of a randomised, double-blind, placebo-controlled, phase 1B/2A trial. Lancet HIV *8*, e568-e580. 10.1016/S2352-3018(21)00157-0.
7. Voysey, M., Costa Clemens, S.A., Madhi, S.A., Weckx, L.Y., Folegatti, P.M., Aley, P.K., Angus, B., Baillie, V.L., Barnabas, S.L., Bhorat, Q.E., et al. (2021). Single-dose administration and the influence of the timing of the booster dose on immunogenicity and efficacy of ChAdOx1 nCoV-19 (AZD1222) vaccine: a pooled analysis of four randomised trials. Lancet *397*, 881-891. 10.1016/S0140-6736(21)00432-3.
8. Sadoff, J., Gray, G., Vandebosch, A., Cardenas, V., Shukarev, G., Grinsztejn, B., Goepfert, P.A., Truyers, C., Fennema, H., Spiessens, B., et al. (2021). Safety and Efficacy of Single-Dose Ad26.COV2.S Vaccine against Covid-19. N Engl J Med *384*, 2187-2201. 10.1056/NEJMoa2101544.
9. Logunov, D.Y., Dolzhikova, I.V., Shcheblyakov, D.V., Tukhvatulin, A.I., Zubkova, O.V., Dzharullaeva, A.S., Kovyrshina, A.V., Lubenets, N.L., Grousova, D.M., Erokhova, A.S., et al. (2021). Safety and efficacy of an rAd26 and rAd5 vector-based heterologous prime-boost COVID-19 vaccine: an interim analysis of a randomised controlled phase 3 trial in Russia. Lancet *397*, 671-681. 10.1016/S0140-6736(21)00234-8.
10. Halperin, S.A., Ye, L., MacKinnon-Cameron, D., Smith, B., Cahn, P.E., Ruiz-Palacios, G.M., Ikram, A., Lanas, F., Lourdes Guerrero, M., Munoz Navarro, S.R., et al. (2022). Final efficacy analysis, interim safety analysis, and immunogenicity of a single dose of recombinant novel coronavirus vaccine (adenovirus type 5 vector) in adults 18 years and older: an international, multicentre, randomised, double-blinded, placebo-controlled phase 3 trial. Lancet *399*, 237-248. 10.1016/S0140-6736(21)02753-7.
